# Irrational use of colistin sulfate in poultry and domestic animals in Nepal-an emerging public health crisis

**DOI:** 10.1016/j.soh.2024.100063

**Published:** 2024-02-05

**Authors:** Sonu Adhikari, Sarita Phuyal, AbdulRahman A. Saied, Asmaa A. Metwally, Krishna Prasad Acharya

**Affiliations:** Department of Animal Breeding and Biotechnology, Agriculture and Forestry University (AFU), Rampur, Chitwan, 44209, Nepal; Central Referral Veterinary Hospital, Tripurehswar, Kathmandu, 45104, Nepal; Ministry of Tourism and Antiquities, Aswan Office, Aswan, 81511, Egypt; Department of Surgery, Anesthesiology, and Radiology, Faculty of Veterinary Medicine, Aswan University, Aswan, 81511, Egypt; Department of Livestock Services (DLS), Hariharbhawan, Lalitpur, 44600, Nepal

**Keywords:** Colistin sulphate, Misuse, Antimicrobial resistance, Nepal

Colistin sulfate is an antibiotic in the polymyxin family that is utilized as a last-resort therapy for septicemia in humans caused by multidrug-resistant infections. However, its indiscriminate use in veterinary practices poses an increasingly significant risk to the development of colistin resistance and its detrimental effects on public health. Colistin is still widely used in animals for treatment, metaphylaxis, prophylaxis, and growth promotion despite being designated a critically important antimicrobial for human medicine by the World Health Organization (WHO) [1]. In this correspondence, our objective is to examine the repercussions stemming from the illicit use of colistin in livestock and poultry in Nepal.

Currently, in Nepal, colistin sulfate is still widely used in animal sectors, particularly the poultry sector, for growth promotion, prophylaxis, and metaphylaxis, despite the ban by the Department of Livestock Services (DLS) on the use of antibiotics as a growth promoter in animal feed back in 2016 [2]. A recent study by the Veterinary Standard and Drug Regulatory Laboratory (VSDRL) in 382 representative farms in Nepal found that colistin sulfate was one of the most commonly used antibiotics after neomycin and doxycycline in poultry farms in Nepal [3]. Recent import data recorded by the Department of Customs supports this observation. The import data indicates that Nepal imported approximately 8,452 kg of colistin sulfate in 2018, which dropped to 956.49 kg in 2019 [4]. This is because DLS has been strictly implementing the policy that prohibits the import of colistin sulfate for use in animal feed as a feed supplement since 2018. However, this data seems underestimated, as there have been reported cases of the illegal import of colistin from India and China [5]. The study by VSDRL identified that around 23% (89/382) of the farmers never consult veterinarians for the use of antibiotics in the poultry farms [3]. Such practice increases the chance that there is either overuse or underuse of antibiotics, including colistin sulfate in poultry farms in Nepal, causing a serious public health concern in Nepal as it heightens the risk of the development of colistin resistance microbes. Tu and his colleagues in China found that the withdrawal of colistin sulfate led to the reduction in the incidence of X4-type plasmid that harbors the *mcr-1* gene, a gene that confers resistance to colistin sulfate [6]. Thus, the use of colistin sulfate is restricted in neighboring countries, including China [7], to limit the development of colistin resistance.

In recent years, resistance to this antibiotic has been reported in animal health sectors, particularly poultry in Nepal [[8], [9], [10]]. For instance, a recent report on active surveillance of antimicrobial resistance in poultry in Nepal by the Central Veterinary Laboratory (CVL) during 2020–2022 found that out of 951 samples tested, 70.25% (668/951) were *Escherichia coli*, of which 20% were resistant to colistin sulfate [5]. Despite the rigorous active surveillance system, it has not yet been reported in humans. This absence of colistin resistance in humans and frequent reports of colistin resistance in poultry birds highlight the potential adverse public health implications from poultry sectors associated with either the transmission of resistance microbes to humans either through the consumption of poultry meat or through the contamination of the environment with colistin-resistant microbes, as there is a common practice of either using undecomposed poultry litter as a fertilizer on farmland or dumping it into nearby poultry farm environments, as previously mentioned by Acharya and his colleagues [11].

Several factors contribute to the irrational usage of colistin sulfate in Nepal. The first and foremost factor is the poor enforcement of the law prohibiting the use of colistin on animals in Nepal and the ineffective monitoring system to check and enforce the ban. Secondly, there is a lack of surveillance of the antibiotics used and antimicrobial resistance in the field conditions. Thirdly, farmers lack awareness about the harmful effects of colistin sulfate misuse in animals and humans, which has led to the indiscriminate use of colistin sulfate in animals, particularly in the poultry sector. Thus, we must be vigilant about the prudent use of colistin in the animal health sector to protect both animal and human health.

Like other nations, Nepal must adopt a multipronged strategy engaging different stakeholders to address the inappropriate use of colistin sulfate. It is necessary to adopt and strictly enforce strict management procedures for using antibiotics in livestock, including precise requirements for dosage, administration, duration of treatment, and regular monitoring and reporting procedures, along with awareness programs aimed at farmers, veterinarians, and other key stakeholders. Additionally, it is essential to pursue probiotics, vaccines, and improved biosecurity measures to reduce disease incidence without relying on antibiotics as the primary solution. Moreover, it is necessary to improve surveillance capabilities, empowering Nepal to embrace the most effective strategies for curtailing the proliferation of antibiotic-resistant bacteria. Ultimately, Nepal needs to collaborate with neighboring countries to enact national and international laws to minimize the international trade of colistin sulfate.

Nepal can forge a healthier and more resilient future by implementing stringent regulations, conducting educational and awareness campaigns, and investing in sustainable farming techniques. The requisite efforts to tackle this issue must extend beyond academia and encompass government-led initiatives in the realm of public health surveillance.

## Data availability statement

All the data is present in the manuscript.

## Funding source

None.

## Declaration of competing interest

We declare no competing interests.
